# Evaluating veterinary *Staphylococcus* β-lactam breakpoint performance: a comparative analysis of individual agents versus oxacillin as a surrogate

**DOI:** 10.1128/jcm.00168-26

**Published:** 2026-06-12

**Authors:** Sarah J. Gefroh, Briena M. Meier, Robert R. Bowden, Daniel R. Evans, Kelli J. Maddock

**Affiliations:** 1 Veterinary Diagnostic Laboratory, North Dakota State University 3323, Fargo, North Dakota, USA; 2 Department of Pathology, Beth Israel Deaconess Medical Center 376938 , Boston, Massachusetts, USA; 3 Minnesota Department of Health 11055, St. Paul, Minnesota, USA; University of California Davis, Davis, California, USA

**Keywords:** *Staphylococcus pseudintermedius*, veterinary, clinical microbiology, microbial sensitivity tests, beta-lactam resistance

## Abstract

*Staphylococcus pseudintermedius* is an important pathogen in companion animals. Accurate detection and reporting of *mecA*-mediated resistance to β-lactam antimicrobials is essential for guiding appropriate antimicrobial treatments. While surrogate agents are used in human diagnostic laboratories to predict β-lactam results in staphylococci, individual β-lactam breakpoints are published for veterinary staphylococci, discouraging the use of a surrogate. Comparative performance of these individual β-lactam breakpoints to predict methicillin-resistance in veterinary *Staphylococcus* species has not been previously evaluated. This study evaluated oxacillin’s accuracy at detecting *mecA* presence and performance as a surrogate for β-lactams in *S. pseudintermedius* from canids and felids. Antimicrobial susceptibility data for *S. pseudintermedius* were obtained from clinical isolates (*n* = 1,914) from veterinary diagnostic laboratories across the United States and Canada and included broth microdilution results for amoxicillin-clavulanate, cefazolin, cefovecin, cefpodoxime, cephalothin, and oxacillin, along with the corresponding presence or absence of *mecA*. Agreement analysis, sensitivities, and specificities were calculated to compare oxacillin performance with individual β-lactams. Oxacillin demonstrated the highest sensitivity for the prediction of *mecA*-mediated resistance among all antimicrobials analyzed. For isolates possessing *mecA*, the proportion showing resistant minimum inhibitory concentrations to β-lactams other than oxacillin was low, ranging between 16.5% and 85%. Additionally, when compared to oxacillin, all β-lactams other than cefovecin exhibited high rates of very major discrepancies, corroborating the significant risk of reporting false susceptibility when testing these antimicrobials. These findings support utilizing oxacillin as a surrogate agent for predicting β-lactam interpretations and emphasize the necessity for clarified guidance in the Clinical Laboratory Standards Institute VET01S document.

**IMPORTANCE:**

Accurate testing and reporting of β-lactam antimicrobials in *Staphylococcus* species are essential for guiding appropriate antimicrobial therapy in companion animals. Our findings show that oxacillin is the most sensitive antimicrobial for detecting *mecA*-mediated resistance when testing *Staphylococcus pseudintermedius*, while most other β-lactams have low sensitivity for *mecA* and unacceptable rates of very major discrepancies when compared to oxacillin. These results highlight the risk of reporting falsely reporting β-lactam results when interpretations are based solely on testing each individual agent. Therefore, oxacillin and cefoxitin should be used as surrogate markers for β-lactam susceptibility, as appropriate for the *Staphylococcus* species, to minimize the risk of reporting false susceptible results. The clarification of these criteria by the Clinical and Laboratory Standards Institute in VET01S will enhance diagnostic accuracy, aid effective antimicrobial stewardship, and improve clinical outcomes in veterinary medicine.

## INTRODUCTION

*Staphylococcus* species are present within multiple animal species as commensal flora and are often significant agents of infection. It is common for staphylococci to acquire resistance to first-line antimicrobials; therefore, antimicrobial susceptibility testing (AST) is necessary to ensure that selected therapies are appropriate for treatment of clinical infections.

Staphylococcal resistance to penicillinase-stable penicillins and other β-lactams is primarily conferred by the *mecA* gene, which encodes altered penicillin-binding proteins (PBP2a/PBP2a′), as well as by less frequently encountered mechanisms, such as *mecC*, β-lactamase hyperproduction, and other penicillin-binding protein modifications (1). Due to the low affinity of altered penicillin-binding proteins for most β-lactams, resistance conferred by *mec* genes is broad-spectrum, affecting the entire β-lactam class, with the exceptions of ceftaroline and ceftobiprole (2).

Susceptibility to an antimicrobial can be determined by testing the bacterium against the antimicrobial itself, or, in some instances, through the use of surrogate agents to infer susceptibility to an antimicrobial or antimicrobial class. Surrogate agent tests are used when an agent of interest cannot be routinely tested, has poor stability, or if the surrogate agent is found to be a more reliable predictor of susceptibility and resistance than the agent of interest (2, 3).

In 2012, the Clinical and Laboratory Standards Institute (CLSI) Subcommittee on Antimicrobial Susceptibility Testing removed staphylococcal breakpoints for β-lactams other than penicillin, cefoxitin, oxacillin, and ceftaroline from the M100 document used to interpret results for bacteria isolated from humans (4). The rationale for this change was that the surrogate agents (penicillin and cefoxitin/oxacillin) more accurately predicted treatment response due to better detection of genotypic resistance than other β-lactam breakpoints, which often produced susceptible test results while the surrogate agents predicted resistance (4). The European Committee on Antimicrobial Susceptibility Testing (EUCAST) likewise recommends testing penicillin and cefoxitin or oxacillin for staphylococci and has never approved clinical breakpoints for β-lactams other than surrogate agents, except for ceftaroline and ceftobiprole (5).

In contrast to CLSI M100 (2) and EUCAST (5), CLSI VET01S (6) currently includes species-specific β-lactam breakpoints for canids, felids, and equids (Table 1) as well as the surrogate agents penicillin, oxacillin, and cefoxitin, using breakpoints published in CLSI M100. Inclusion of both species-specific β-lactam breakpoints and M100-defined surrogate agent recommendations suggests that each should be considered for routine testing and creates confusion as to which should be given precedence when results are discordant.

**TABLE 1:** VET01S 7th edition breakpoints for *Staphylococcus* spp.

Drug	Organism	Susceptible	Intermediate	Resistant	Breakpoint source species
		MIC (µg/mL)			
Amoxicillin-clavulanate	*Staphylococcus* spp.	≤0.25/0.12	0.5/0.25	≥1/0.5	Canid and felid
Cefazolin	*S. aureus* and *S. pseudintermedius*	≤2	4	≥8	Canid
Cefovecin	*S. pseudintermedius*	≤0.5	1	≥2	Canid
Cefpodoxime	*S. aureus* and *S. pseudintermedius*	≤2	4	≥8	Canid
Cephalothin	*S. aureus* and *S. pseudintermedius*	≤2	4	≥8	Canid
Oxacillin	*S. pseudintermedius* and *S. schleiferi*	≤0.25	NA^*b*^	≥0.5	Human^*a*^

^*a*^ Human oxacillin breakpoint published in CLSI VET01S, 7th edition originates from a previous version of M100 and does not reflect current M100 breakpoints.

^*b*^ NA, not applicable.

Harmonizing staphylococcal AST guidelines and reporting practices between human and veterinary diagnostics could offer significant benefits. From a laboratory perspective, removing extra β-lactam breakpoints can eliminate redundancy in testing and minimize errors in reporting. Providers can expect more succinct reports, improved clarity due to a lack of contradictory AST results, and ultimately, results that more accurately predict treatment response. Additionally, AST device manufacturers will be able to reduce the number of β-lactams on their devices, allowing room for new antimicrobials, antimicrobials of public health concern, updated dilution ranges to cover new or revised breakpoints, and expanded dilution ranges that enable improved quality control (QC) testing.

The correlation of *mecA* with oxacillin results for *S. pseudintermedius* is well-established (7–10). The objective of this study was to evaluate performance of CLSI VET01S canine-specific β-lactam breakpoints for *Staphylococcus pseudintermedius* against oxacillin surrogate agent interpretive criteria, identifying which testing strategy most reliably detects the presence of *mecA* and whether surrogate testing could replace testing each individual β-lactam agent.

## MATERIALS AND METHODS

### Bacterial isolates

*S. pseudintermedius* isolates (*n* = 5,866) were collected from canine clinical samples submitted for diagnostic testing to veterinary diagnostic laboratories located across the United States (U.S.) and Canada as part of the U.S. Food and Drug Administration Veterinary Laboratory Investigation and Response Network Antimicrobial Resistance (FDA Vet-LIRN AMR) Monitoring Program from 2017 to 2022 (11). AST data collection for this program has been described previously (10, 12). Two thousand twenty-four of these isolates had whole-genome sequencing (WGS) data available through the National Library of Medicine (13), and of those, 1,914 had minimum inhibitory concentration (MIC) values that could be interpreted for all agents analyzed in this study using the current VET01S (6) *S. pseudintermedius* breakpoints (Table 1). Isolates without WGS data or those with MIC values that could not be interpreted for one or more agents based on these breakpoints (e.g., oxacillin MIC of ≤1) were excluded from this analysis.

### Antimicrobial susceptibility testing

All isolates in this study were tested by participating laboratories using commercial freeze-dried broth microdilution (BMD) AST panels. Isolates in this study (1,914/1,914; 100%) were tested using Sensititre COMPGP1F or COMPAN2F panels (ThermoFisher Scientific, Lenexa, KS, USA). Isolates with MICs that could be interpreted based on current CLSI veterinary breakpoints (Table 1) for amoxicillin-clavulanate, cefazolin, cefovecin, cefpodoxime, cephalothin, and oxacillin (*n* = 1,914) were evaluated; these agents are collectively referred to as β-lactams throughout this study. Oxacillin disk diffusion (6) was performed by North Dakota State University Veterinary Diagnostic Laboratory on selected isolates (*n* = 80) with discrepancies between the genotypic and phenotypic AST methods.

### Data retrieval

Raw AST data were retrieved from the FDA Vet-LIRN AMR Database (11). The corresponding *mecA* genotype identified by the AMRFinderPlus (14) application for each accession was downloaded from the National Center for Biotechnology Information (NCBI) Pathogen Detection (13) (BioProject PRJNA314609) on 20 March 2025, for analysis.

### Interpretive criteria

AST MIC data were interpreted based on breakpoints published in the CLSI VET01S 7th Edition (6) (Table 1). Oxacillin disk diffusion results were interpreted using VET01S 7th edition criteria (zone diameters ≥ 18 mm = susceptible; ≤17 mm = resistant) (6).

### Discrepancy rates and categorical agreement

Discrepancy and agreement rates were calculated to assess the relationship between the surrogate and comparison agent, as previously described. Very major discrepancies (VMDs) were defined in this study as isolates that tested susceptible to the comparison agent (amoxicillin-clavulanate or a cephalosporin) but tested resistant to the surrogate agent (oxacillin). Major discrepancies (MDs) were defined as isolates that tested resistant to the comparison agent but tested susceptible to the surrogate agent. When interpretive differences between a comparison and surrogate agent occurred, gene presence or absence was used as the reference method for discrepancy resolution. Categorical agreement (CA) between surrogate and comparison agents was calculated as the total number of isolates testing either susceptible to both antimicrobials or not susceptible (resistant or intermediate) to both antimicrobials, divided by the total number of isolates with interpretations for both antimicrobials.

### Test performance metrics

Calculations used to assess the correlation between resistance markers and antimicrobials included sensitivity, specificity, positive predictive values (PPVs), and negative predictive values (NPVs). Gene presence/absence was compared to categorical AST results for each antimicrobial (susceptible, intermediate, or resistant). Sensitivity calculations included all isolates with *mecA* that were not susceptible to the antimicrobial, including isolates testing intermediate.

### Additional genotypic concordance assessment

Whole-genome assemblies were downloaded from the NCBI database to detect genetic markers of antimicrobial resistance (AMR). Genomes were queried for acquired AMR genes deposited in the Comprehensive Antimicrobial Resistance Database (CARD), NCBI AMR, and ResFinder repositories with ABRicate v1.2.0, using default program settings and databases updated as of 8 January 2026 (14–18).

## RESULTS

### Prevalence of *mecA*

Of the 1,914 isolates included in this study, 32.0% (*n* = 613) were positive for *mecA*.

### Correlation of β-lactam results with presence or absence of *mecA*

Isolates were categorized according to genotype and then sorted based on AST interpretation (Tables 2 and 3). With these data, the performance of oxacillin and each of the β-lactams (amoxicillin/clavulanate, cefazolin, cefovecin, cefpodoxime, and cephalothin) for the prediction of *mecA* presence or absence was assessed (Table 4). Of the antimicrobials evaluated, oxacillin resistance correlated most consistently with *mecA* presence, with a sensitivity of 95.6% (*n* = 586/613) (Table 4). Overall, only oxacillin and cefovecin demonstrated a *mecA* detection sensitivity of ≥90%, while other antimicrobials had sensitivities between 22.0% and 83.7%. All antimicrobials achieved specificities of 95% or higher.

**TABLE 2:** Isolates with *mecA* categorized by antimicrobial interpretation^*a*^

		S	I	R
Antimicrobial	*N*	% (*n*)		
Amoxicillin/clavulanate	613	16.3 (100)	27.9 (171)	55.8 (342)
Cefazolin	613	70.6 (433)	7.3 (45)	22.0 (135)
Cefovecin	613	6.0 (37)	8.6 (53)	85.3 (523)
Cefpodoxime	613	27.7 (170)	11.3 (69)	61.0 (374)
Cephalothin	613	80.0 (478)	5.5 (34)	16.5 (101)
Oxacillin	613	4.4 (27)	NA	95.6 (586)

^*a*^ Abbreviations: I, intermediate; R, resistant; S, susceptible; NA, not applicable.

**TABLE 3:** Isolates without *mecA* categorized by antimicrobial interpretation^*a*^

		S	I	R
Antimicrobial	*N*	% (*n*)		
Amoxicillin/clavulanate	1,301	96.6 (1257)	1.8 (24)	1.5 (20)
Cefazolin	1,301	98.1 (1276)	0.5 (6)	1.5 (19)
Cefovecin	1,301	95.6 (1244)	1.2 (15)	3.2 (42)
Cefpodoxime	1,301	97.0 (1262)	0.9 (12)	2.1 (27)
Cephalothin	1,301	98.0 (1275)	0.6 (8)	1.4 (18)
Oxacillin	1,301	95.4 (1241)	NA	4.6 (60)

^*a*^ Abbreviations: I, intermediate; R, resistant; S, susceptible; NA, not applicable.

**TABLE 4:** Performance of oxacillin and select β-lactams for prediction of *mecA* presence or absence^*a*^

	Sensitivity	Specificity	PPV	NPV
	% (*n*/*N*)			
Amoxicillin/clavulanate	83.7 (513/613)	96.6 (1,257/1,301)	92.1 (513/557)	92.6 (1,257/1,301)
Cefazolin	29.4 (180/613)	98.1 (1,276/1,301)	87.8 (180/205)	74.7 (1,276/1,709)
Cefovecin	94.0 (576/613)	95.6 (1,244/1,301)	90.1 (576/633)	97.1 (1,244/1,281)
Cefpodoxime	72.2 (443/613)	97.0 (1,262/1,301)	91.9 (443/482)	88.1 (1,262/1,432)
Cephalothin	22.0 (135/613)	98.0 (1,275/1,301)	83.9 (135/161)	72.7 (1,275/1,753)
Oxacillin	95.6 (586/613)	95.4 (1,241/1,301)	90.7 (586/646)	97.9 (1,241/1,268)

^*a*^ Abbreviations: NPV, negative predictive value; PPV, positive predictive value.

There were 1,241 isolates that were susceptible to oxacillin and negative for the *mecA* gene, with 97.9% (*n* = 1,215/1,241) demonstrating susceptible results for all other tested β-lactam antibiotics (Table 5). Of the remaining 2.1%, 20 isolates tested as intermediate to a β-lactam. The remaining 6 discrepant isolates were resistant to one or more of the β-lactams, accounting for 0.5% of all oxacillin-susceptible isolates lacking *mecA*.

**TABLE 5:** Oxacillin-susceptible isolates without *mecA,* categorized by antimicrobial interpretation^*a*^

Antimicrobial	*N*	S	I	R
Amoxicillin/clavulanate	1,241	1,236	5	0
Cefazolin	1,241	1,235	4	2
Cefovecin	1,241	1,231	6	4
Cefpodoxime	1,241	1,234	6	1
Cephalothin	1,241	1,235	5	1

^*a*^ Abbreviations: I, intermediate; R, resistant; S, susceptible.

Eighty-seven isolates with discrepancies between oxacillin interpretation and mecA results were identified, with 80 of these isolates available and obtained for further investigation by disk diffusion testing. These results can be found in Tables S1 to S3 (see Supplemental Material). Of 56 mecA-negative isolates that initially tested resistant to oxacillin by BMD, 75% (*n* = 42/56) revealed susceptible results when retested by disk diffusion, with 14 isolates remaining discrepant. Likewise, of the 24 mecA-positive isolates that initially tested susceptible to oxacillin by BMD, 41.7% (*n* = 10/24) were found to be resistant when retested by disk diffusion, with 14 isolates remaining discrepant.

### Additional genotypic concordance assessment

Additional genomic analyses were performed on 54 isolates with unresolved genotypic-phenotypic discordant results. These isolates consisted of 26 that were *mecA*-negative and oxacillin-susceptible but showed intermediate or resistant results to another β-lactam, 14 isolates that were *mecA*-negative but oxacillin-resistant, and 14 that were *mecA*-positive but oxacillin-susceptible. One genome was found to be *S. aureus* improperly labeled as *S. pseudintermedius* in Pathogen Detection, and two genomes were flagged as contaminated, potentially accounting for the discordance between genotype and phenotype for these isolates. No *mecA* homologs (*mecB* and *mecC*) were identified in any isolates.

### Categorical agreement between oxacillin and other β-lactams

Using the breakpoints listed in Table 1, the CA, MD, and VMD rates between oxacillin and the other β-lactams were calculated, with genotype (presence or absence of *mecA*) as the gold standard for discrepancy resolution (Table 6). Cefovecin had the highest CA with oxacillin at 98.8% (*n* = 1,891/1,914), with CA ranging from 76% to 92.2% for the other β-lactams. The VMD rate for cefovecin was 1.9% (*n* = 12/646) and >10% for all other antimicrobials. MD rates ranged from 0.1% to 0.3%.

**TABLE 6:** Performance of other β-lactams compared with oxacillin results

Drug	Total no. of isolates tested	CA % (*n*)^*a*^	VMD % (*n*/*N*)^*b*^	MD % (*n*/*N*)^*c*^
Amoxicillin/clavulanate	1,914	95.8 (1,833)	11.6 (75/646)	0 (0/1,268)
Cefazolin	1,914	78.5 (1,502)	62.8 (406/646)	0.2 (2/1,268)
Cefovecin	1,914	98.8 (1,891)	1.9 (12/646)	0.3 (4/1,268)
Cefpodoxime	1,914	92.2 (1,764)	22.1 (143/646)	0.1 (1/1,268)
Cephalothin	1,914	76.0 (1,455)	70.0 (452/646)	0.1 (1/1,268)

^*a*^ CA = Categorical agreement reflects the number of isolates with no discrepancies out of the total number of isolates tested.

^*b*^ VMD = Very major discrepancies are isolates testing susceptible to the comparison agent (amoxicillin-clavulanate or cephalosporin) and resistant to the surrogate agent (oxacillin).

^*c*^ MD = Major discrepancies are isolates testing susceptible to the surrogate agent, and resistant to the comparison agent.

### Differences in oxacillin breakpoints between CLSI standards

Due to differences between oxacillin breakpoints for *S. pseudintermedius* in VET01S and M100 (≥0.5 µg/mL resistant and ≥1 µg/mL resistant, respectively), an investigation of *mecA* presence was performed on isolates with MICs of 0.5 or 1 µg/mL. 84.9% (*n* = 90/106) of isolates with oxacillin MICs of 0.5 µg/mL and 91.2% (*n* = 115/126) of isolates with MICs of 1 µg/mL were positive for *mecA*.

## DISCUSSION

The *mecA*-mediated resistance mechanism is universal in staphylococci infecting humans and animals alike, and detection of this acquired resistance is the primary goal of staphylococcal β-lactam testing. In this study, we compared the performance of individual β-lactam breakpoints for *S. pseudintermedius* with oxacillin as a surrogate marker for *mecA*-mediated resistance. AST and genotypic data from a large collection of isolates representing a diverse geographical distribution demonstrated oxacillin to be the most sensitive phenotypic indicator of *mecA* presence among the antimicrobials evaluated. Further analysis concluded that oxacillin susceptibility was predictive of susceptibility to other β-lactam agents. Conversely, many individual β-lactam results significantly underestimated *mecA*-mediated resistance in *S. pseudintermedius*, revealing the risk of reporting false-susceptible results if using these breakpoints and supporting the use of oxacillin as a surrogate agent for β-lactam reporting in veterinary diagnostics.

Oxacillin demonstrated a high sensitivity and specificity for *mecA* (95.6% and 95.4%, respectively). Oxacillin also accurately predicted susceptibility to other β-lactams 97.6% of the time, emphasizing its reliability as a surrogate for β-lactam testing. Of the few instances where oxacillin susceptibility did not correlate with susceptibility to the comparison agents, 23 *mecA*-negative isolates were oxacillin susceptible but had intermediate interpretations for one or more β-lactams. Because the comparison agent intermediate values are within 1 dilution of the susceptible breakpoint, these discrepancies could be due to routine testing variability of ±1 doubling dilution that impacts categorical interpretation for isolates with MICs that fall close to the interpretive breakpoints. There were also six unique isolates with resistance to a β-lactam that did not correlate with *mecA* presence or oxacillin resistance. These errors might be attributable to β-lactamase hyperproduction, a non-*mec*-mediated resistance gene, or PBP mutation, analytical error, or testing anomaly. However, as repeat MIC testing was not performed and isolated resistance to a single β-lactam in *mecA*-negative isolates lacks a known, consistent resistance mechanism in *S. pseudintermedius*, this lends more credibility to the six observed discrepancies representing MIC test performance errors.

Isolates with a disagreement between the oxacillin phenotype and *mecA* genotype were rare. Approximately half of the oxacillin-susceptible and *mecA*-positive discrepancies were resolved with supplemental disk diffusion testing demonstrating resistance. Additional genotypic analyses did not identify *mecA* homologs. The remaining number of discrepancies was small compared to the total number of isolates analyzed in this study (*n* = 14/1,914), possibly representing either analytical errors, or isolates with unexpressed, or low-level, or heterogeneous expression of *mecA*. Conversely, 14 of the 56 isolates with resistant oxacillin MICs and absence of the *mecA* gene were confirmed to be resistant to oxacillin by disk diffusion, suggesting that a very small subset of isolates have the potential to display oxacillin resistance due to alternate resistance mechanisms, potentially *blaZ* hyperproduction or altered penicillin-binding proteins (termed borderline oxacillin-resistant *S. aureus* [19] and modified *S. aureus* [20], respectively, within the context of *S. aureus*). Definitive explanations for isolates displaying disagreement between phenotypic and genotypic results are beyond the scope of this study but would be of interest for future investigation. Ultimately, the rate of discordance between oxacillin and *mecA* results was low in this study, emphasizing the strength of oxacillin as a reliable surrogate to accurately predict susceptibility and resistance to the included β-lactams.

When considering the utility of oxacillin as a surrogate agent, it should be noted that revised oxacillin MIC breakpoints for *S. pseudintermedius* were published in CLSI M100 beginning in 2021 (21), with the breakpoints adjusted from ≤0.25 µg/mL susceptible and ≥0.5 µg/mL resistant to ≤0.5 µg/mL susceptible and ≥1 µg/mL resistant. The VET01S (6) has avoided implementing these changes on the basis that *mecA*-positive isolates will be miscategorized. This perspective is supported by the present study’s data. Although a higher specificity for *mecA* was observed for isolates with an MIC of 1 µg/mL as compared to those with an MIC of 0.5 µg/mL, applying the new M100 breakpoints would come at a high cost in the form of lower detection sensitivity. As 84.9% of isolates in the present study with oxacillin MICs of 0.5 µg/mL were *mecA*-positive, it would seem best for patient care to continue categorizing this MIC as resistant rather than susceptible.

In the evaluation of the comparison agents, all except cefovecin demonstrated an unacceptably high VMD rate (11.6–70%) compared to oxacillin, exceeding what would be considered standard acceptance criteria in a test method validation. This indicates a substantial risk of false-susceptible reporting if individual β-lactam breakpoints are used. Correlation of β-lactams with *mecA* demonstrated poor sensitivities between 22% and 83.7%, except for cefovecin. While cefovecin had a low VMD (1.9%) and high sensitivity (94%) for *mecA* detection (when including both intermediate and resistant results), further investigation showed that 15% of *mecA*-positive isolates tested susceptible or intermediate to cefovecin, illustrating that even a high-performing β-lactam provides poorer guidance than is obtained by testing oxacillin. These factors should prompt laboratories to utilize oxacillin as a surrogate to interpret cephalosporin and β-lactam results for *Staphylococcus pseudintermedius* to avoid inadvertently producing misleading AST reports.

Among the agents evaluated, the oxacillin NPV is the highest (97.9%), indicating its reliability for ruling out infections by *mecA*-positive *S. pseudintermedius*. This high NPV may support more conservative prescribing practices, such as the use of a first-generation cephalosporin, in cases with oxacillin-susceptible results. Conversely, the oxacillin PPV (90.7%) reaffirms the ability of oxacillin to accurately predict *mecA*. As demonstrated in this analysis, use of individual β-lactam AST interpretations offers less reliable guidance for veterinarians, as multiple agents show poor correlation with *mecA*-mediated resistance. Collectively, this evidence supports veterinary utilization of oxacillin as a surrogate agent to improve the accuracy of AST reports, aid diagnostic clarity for providers, and strengthen stewardship efforts.

We hope that the implementation of the proposals presented here may improve clinical practice, as treatment guidance for veterinarians has become less clear in recent years. In 2014, the International Society for Companion Animal Diseases (ISCAID) stated “Meticillin (oxacillin)-resistant staphylococci are by convention resistant to all β-lactam AMDs (antimicrobial drugs) (cephalosporins, penicillins, carbapenems, and monobactams), regardless of occasional apparent *in vitro* susceptibility” for superficial bacterial folliculitis (22). However, the 2025 ISCAID pyoderma guidelines recommend testing amoxicillin-clavulanate, cephalothin (or cefazolin), and cefpodoxime, while also stating that methicillin-resistant staphylococci are by convention resistant to all β-lactams and should be reported as such, regardless of *in vitro* results (23). Furthermore, they suggest that cephalexin may be appropriate for treatment of “low-level“ methicillin-resistant *S. pseudintermedius* (MRSP) if MICs for that antimicrobial indicate susceptibility (23). This update seems to be largely derived from a single paper, which included *in vitro* data as well as a small case series (24). While interesting, the paper had significant confounders, such as small sample size, concurrent adjunctive topical therapy, and/or long treatment durations, making it difficult to determine if the clinical outcomes were directly linked to β-lactam MIC. Additionally, the reported cephalexin MIC distribution data showed a modal MIC value two doubling dilutions lower than is typical even for isolates that lack *mecA*, suggesting the possibility of AST methodological issues. Furthermore, it has previously been demonstrated that commercial AST devices may produce lower oxacillin MICs for MRSP than are observed when the same isolates are tested using frozen reference BMD panels (9). This suggests that high-level oxacillin resistance may appear as low-level resistance using the methods commonly employed in veterinary diagnostics laboratories, and that MIC results for β-lactams other than oxacillin might also be lower when tested by certain commercial methods. When all these factors are considered together, it does not currently seem justifiable to routinely report *mecA*-positive isolates as susceptible to any β-lactam agents used in veterinary medicine.

A limitation of this study is that MICs were determined using commercial AST devices and not the reference method BMD. However, this method is routinely used in veterinary diagnostic laboratories, and these data demonstrate that real-world application of the veterinary breakpoints for β-lactams other than surrogate agents exhibits unacceptable performance and poor correlation with *mecA* and/or oxacillin results. While our data set was focused solely on *S. pseudintermedius* isolated from dogs, we believe there is sufficient literature regarding *mecA* and correlation with oxacillin and cefoxitin (2, 4, 5, 9, 25) to reach the conclusion that surrogate agents would be appropriate predictors of β-lactam susceptibility and *mecA*-mediated resistance detection for all staphylococci isolated from animal species.

The findings of this study support previous literature (4) that β-lactam combination agents and cephalosporin breakpoints carry significant risk of reporting false-susceptible results that could undermine both clinical outcomes and stewardship efforts. Due to the higher discrepancy rates observed for the comparison β-lactam agents with veterinary breakpoints, it seems inadvisable to use individual CLSI VET01S canine staphylococcal breakpoints for amoxicillin-clavulanate, cefazolin, cefovecin, cefpodoxime, cephalexin, and cephalothin, as well as the feline breakpoints for amoxicillin-clavulanate. These data were presented to the CLSI Veterinary Antimicrobial Susceptibility Testing (VAST) Subcommittee in January of 2026, and our recommendations to remove veterinary-specific β-lactam breakpoints were unanimously approved (26). This update, tentatively expected to be published in the 8th edition of VET01S, will retain the current VET01S *S. pseudintermedius* susceptible (≤0.25 µg/mL) and resistant (≥0.5) breakpoints for oxacillin. While some staphylococcal breakpoints may require review, the document will ultimately retain other established staphylococcal oxacillin and cefoxitin breakpoints that align with M100 (2) and EUCAST (5). To reduce confusion about the appropriateness of the surrogate agent breakpoints, these breakpoints will no longer be labeled as “human” and will be clearly indicated for use in all veterinary species. Ideally, veterinary AST devices will soon be updated to exclude individual β-lactams other than penicillin, ampicillin, oxacillin, and cefoxitin. Together, these changes will simplify testing and reporting, increase the accuracy of results, and enhance clinical decision-making to improve patient outcomes. As antibiotic resistance continues to rise, now is the time for greater coordination between the One Health sectors, aligning our efforts against the pathogens that impact us all.

## SUPPLEMENTAL MATERIAL

Tables S1 to S3 jcm.00168-26-s0001.xlsx Correlation of isolates with phenotypic results.

ASM does not own the copyrights to Supplemental Material that may be linked to, or accessed through, an article. The authors have granted ASM a non-exclusive, world-wide license to publish the Supplemental Material files. Please contact the corresponding author directly for reuse.
